# Trend in magnitude of tuberculosis in Awi Zone, Northwest Ethiopia: a five-year tuberculosis surveillance data analysis

**DOI:** 10.1186/s13104-019-4234-z

**Published:** 2019-04-05

**Authors:** Tefera Alemu, Hordofa Gutema

**Affiliations:** 1 Amhara Public Health Institute, Dessie, Ethiopia; 2 Department of Health Promotion and Behavioural Sciences, School of Public Health, College of Medicine and Health Sciences, Bahir Dar University, Bahir Dar, Ethiopia

**Keywords:** Trend, Magnitude, Tuberculosis, Awi Zone

## Abstract

**Objective:**

Ethiopia is among the 30 high tuberculosis (TB) burden countries with annual estimated TB incidence of 164/100,000 population and death rate of 28/100,000 population for 2017. We analyzed the trend in magnitude of tuberculosis in Awi Zone from July 08/2011 up to June 27/2016.

**Results:**

Tuberculosis surveillance data (2012 to 2016) was extracted from Awi Zonal Health Department Health Management Information System database and TB program unit and analyzed by Microsoft Excel 2007^®^. Epi-Info^7^ software was used for tuberculosis trend analysis using Chi square for trends. A total of 8193 new TB cases were included in the analysis, of which 18.7% were smear positive PTB cases, 28.5% smear negative PTB (PTB−) cases and 52.7% were extra pulmonary TB (EPTB) cases. All form TB prevalence rate was 213/100,000 population in 2012 and significantly decreased to 189 in 2016 (Trend χ^2^ = 11.97; P = 0.00054). Similarly, all form TB incidence rate was 167/100,000 population in 2012 and decreased to 122 in 2016 (Trend χ^2^ = 37.6; P = 0.000). Overall, the magnitude of tuberculosis had decreased over the periods reviewed. The proportion of EPTB is high. We recommend culture and chest X-ray diagnostic services expansion to capture EPTB and PTB− cases.

## Introduction

Tuberculosis (TB) is a chronic infectious disease caused by Mycobacterium tuberculosis. It typically affects the lungs (pulmonary TB) but can affect other parts of the body as well (extra pulmonary TB). The disease is spread via droplet infection when people with pulmonary TB expel the bacilli while coughing, sneezing, talking, etc. Without treatment, mortality rates are high [1].

About a quarter of the world’s population is estimated to be infected with tubercle bacilli and hence millions of people are at risk of developing active disease each year [2]. According to World Health Organization 2018 global TB report, an estimated 10 million people have developed TB disease in 2017, of which 5.8 million were men, 3.2 million among women and 1 million were children. Overall, 90% were adults and 9% were people living with HIV. There were also an estimated 1.3 million TB deaths in 2017, and an additional 0.3 million deaths resulting from TB disease among HIV-positive people [3].

Ethiopia is among the 30 high tuberculosis, human immunodeficiency virus and multidrug resistance tuberculosis burden countries that accounted for 87% of all estimated TB cases worldwide with annual estimated TB incidence of 164/100,000 population and death rate of 28/100,000 population for 2017 [4].

Therefore, analysis of any data is the backbone of interpreting any public health raw data; and as being in the public health domain TB data is also in need to be interpreted as of other data as well since it is one of the public health concern in Ethiopia. In this regard there is no quality information on the existing tuberculosis burden and trend of TB disease in the area so far. Therefore, the aim of this study was to determine the trend in magnitude of tuberculosis in Awi administrative zone, Northwest Ethiopia.

## Main text

### Methods

Study setting and period: Awi Zone is located in western parts of Amhara Region and it is bordered on the west by Benishangul-Gumuz Region, on the north by West Gondar Zone and on the east by West Gojjam Zone. The zone is located at a distance of 114 km from Bahir Dar and 449 km from Addis Ababa. Based on the 2007 census data conducted by Central Statistical Agency, this zone has a total population of 1,220,316 in 2016, of whom 598,880 (49.1%) are men and 621,436 (50.9%) are women [5]. In the study area TB prevention and control program is coordinated by TB program officers at the district level who lead the implementation of TB program across their respective cluster health centres and health posts. There are, on average one health centre and five satellite health posts for an estimated 25,000 population. Each health centre has a designated TB clinic, which provides TB diagnostic and treatment services and managed by a full-time and trained TB focal person. Each health post which is staffed by at least two health extension workers serves as DOTS sites and undertakes active community TB surveillance, tuberculosis treatment defaulter tracing and awareness creation in the community. Partners also actively collaborate with government in TB program implementation in the area. The study was conducted in December 2016.

- *Study design*: A retrospective record review method was employed to extract data on Tuberculosis indicators.
- *Data collection and analysis procedure*: Five**-**year TB secondary data (2012 to 2016) was extracted from Awi Zonal Health Department Health Management Information System (HMIS) database and TB program unit. Furthermore, data cleaning was done together with TB program officers and finally data analysis was done using Microsoft Office Excel 2007 and results was presented by simple descriptive and frequency tables and graph. Moreover, tuberculosis trend analysis was done using Chi square for trends with Epi Info^7^ software.

TB cases classification

- *Smear-positive pulmonary TB (PTB+)*: a patient with at least two initial sputum smear examination positive for acid fast bacilli (AFB) by direct microscopy. Or one initial smear examination positive and culture positive or one initial smear positive and radiological abnormalities [6].
- *Smear-Negative TB (PTB*−*)*: a patient having symptoms suggestive of TB with at least three initial smear examinations negative for AFB by direct microscopy or positive by culture [6].
- *Extra-pulmonary TB (EPTB)*: TB in organs other than the lungs, proven by one culture positive specimen from an extra- pulmonary site or histo-pathological evidence from biopsy or strong clinical evidence consistent with active EPTB (by decision of clinician [6].

Definition of indicators

- *All form TB*: All types of Tuberculosis; i.e. pulmonary positive, pulmonary negative & extra pulmonary tuberculosis [6].
- *Cured*: An initially smear-positive patient who is sputum smear-negative at, or one ‘month’ prior to, the completion of treatment [6].
- *Treatment completed*: A patient who completed treatment but for whom smear results are not available at 5th or 7th month or 1  month prior to the completion of treatment [6].
- *Died*: A patient who dies for any reason during the course of treatment of tuberculosis [6].
- *Treatment success*: The sum of patients who are declared “cured” and those who have “completed” treatment [6].

### Results

In the study period, a total of 8193 all form TB cases (incident) were officially notified to Awi Zonal Health Department with 53.3% (4364) being males. Regarding the age distribution of cases, 2.5% (202) were children less than 5 years, 10.2% (835) were 5 up to 15 years and 87.3% (7156) were adults aged greater than 15 years old. The average proportion of TB forms in the zone was found to be 18.7%, 28.5% and 52.8% for PTB+ , PTB− and EPTB respectively. There is no temporal increase or decrease in the proportion of patients with EPTB; rather we found a consistently high proportion of EPTB cases throughout the study periods. The annual number of all form TB cases has declined significantly from 1801 cases in 2012 to 1491 cases in 2016 with a downward trend in the odds ratios (Trend χ^2^ = 37.6; P = 0.000) (Table 1).

**Table 1:** Trend in TB notifications, Awi Zone, Northwest Ethiopia, 2012-2016 (N=8193)

Year	PTB+ [N (%)]	PTB− [N (%)]	EPTB [N (%)]	All form TB [N (%)]
2012	345 (19.1)	527 (29.3)	929 (51.6)	1801 (100)
2013	344 (19.1)	514 (28.6)	941 (52.3)	1799 (100)
2014	295 (20.1)	453 (30.9)	720 (49)	1468 (100)
2015	264 (16.1)	516 (31.6)	854 (52.3)	1634 (100)
2016	288 (19.3)	326 (21.9)	877 (58.8)	1491 (100)
Total	1536 (18.7)	2336 (28.5)	4321 (52.8)	8193 (100)

**Fig. 1:**
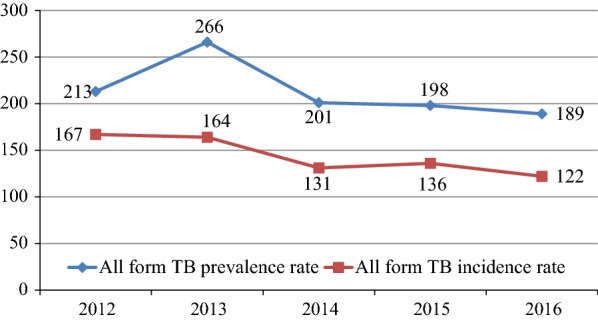
Trend in prevalence and incidence rate of all form TB per 100,000 population in Awi Zone, Northwest Ethiopia, 2012–2016

Average prevalence and incidence rate of all form TB in the zone was 213 and 144 per 100,000 population respectively. The prevalence rate of all form TB was as high as 266 in 2013 and as low as 189 in 2016 with a downward trend in the odds ratios, especially between 2013 and 2014 (Trend χ^2^ = 11.97; P = 0.00054). Similarly, the incidence rate of all form TB was 167 in 2012 and showed consecutive decrement across the study periods to reach 122 in 2016 (Trend χ^2^ = 37.6; P = 0.000) (Fig. 1).

Average prevalence and incidence rate of smear positive pulmonary TB in the zone was 41 and 27 per 100,000 population respectively. The prevalence rate of smear positive pulmonary TB was high in 2013 and showed significant decrement in the consecutive years (Trend χ^2^ = 22.07; P = 0.000). Similar decreasing trend has been seen in smear positive pulmonary TB incidence rate across the years (Trend χ^2^ = 12.3; P = 0.00045). Treatment success rate was above 90% throughout the study periods and the trend in smear positive TB cure rate was decreased from 74.6% in 2012 to 70.9% in 2013 and then steadily increased to 91.3% in 2016. Overall, the average treatment success and cure rate was 93.1 and 79.2% respectively. The average death rate in this study is nearly 237 (3%). Despite its irregularity the overall trend in death rate showed an increasing trend over the study years (Trend χ^2^ = 11.86; P = 0.00057) (Table 2).

**Table 2:** Trend in tuberculosis magnitude and treatment outcomes in Awi Zone, Northwest Ethiopia, 2012-2016

Year	PTB+ prevalence rate/100,000	PTB+ incidence rate/100,000	Treatment success rate (%)	Cure rate (%)	Death rate [N (%)]
2012	47	32	93.6	74.6	44 (2.4)
2013	50	31	92.3	70.9	22 (1.2)
2014	39	26	94.6	73.9	63 (4.3)
2015	35	22	91.7	85.3	54 (3.3)
2016	34	24	93.2	91.3	54 (3.6)

### Discussion

Majority (87.3%) of the cases were among people aged greater than 15 years old while children aged less than 5 years and 5 to 15 years comprised of 2.5% and 10.2% respectively. This finding is in line with the belief that tuberculosis is a disease of adults and comparable with the 2011 national TB prevalence survey finding and the report from northwest Ethiopia [1, 7]. Similar age distribution was also noted in related studies of neighboring countries [8]. In most countries the proportion of TB were found to be higher for men than women even in countries with equal access to health care service [9–12]. However, in the present study we didn’t found significant difference in the proportion of TB among men and women, which is consistent with the finding from Zambia [13].

The proportion of TB forms as of the analysis is 18.7%, 28.5% and 52.7% for PTB+, PTB− and EPTB respectively. This finding is comparable with the report of a related study from northwest (19.3%, 28.5% and 51.3%) and western (21.5%, 34% and 44.4%) parts of the country respectively [7, 14]. Similar findings were also reported from previous studies in Ethiopia [15–17]. However, the 18.7% prevalence of smear positive TB in this study is far from the 58% recent WHO estimate for Ethiopia [4]; the 50.3% and 59.6% reports from southeast and northwest Nigeria [8, 18]. Moreover, in the present study more than half (52.7%) of TB patients were EPTB cases; which is paradoxical with the theoretical sciences that smear positive pulmonary TB is the predominant form of tuberculosis. And also, the high proportion of EPTB cases observed in our study disagrees with the reports of a related studies from India (30.5%), Baltimore city of the United States (28.2%), Gambella Region of Ethiopia (27.5%), and eastern Sudan (26.6%) [11, 12, 19, 20]. This can be partly explained by the difference between EPTB, PTB+, and PTB^_^ patients with respect to their HIV status, age range, residency, gender and race; however, it is also possible that our finding suggests epidemiological shift towards EPTB. Nonetheless, further research is warranted to investigate the reasons for the high proportion of EPTB cases in our study area.

The average smear positive TB prevalence rate (41/100,000) in the present study is lower than the 2011 national TB prevalence report of 63/100,000 population [1], 78 and 80/100,000 population reported in rural district of Ethiopia [21, 22], the 249/100, 000 adult population in Tanzania [23] and the estimated 228/100,000 population in southern India [10]. However, this finding is higher than the finding of TB prevalence survey, 30/100,000 population done in southwest Ethiopia [24]. Moreover, in the present study average prevalence rate of all form TB was 213/100,000 population. This finding is low compared to the recent national TB prevalence report of Ethiopia (240/100,000), Kenya (426/100,000) and Uganda (253/100,000) [1, 25, 26]. As shown in Fig. 1 after 2013, all form TB prevalence rate showed a steady decline to a prevalence rate of 189/100,000 population in 2016. Also, after 2013 similar decreasing pattern has been noted in the incidence rate of both smear positive and all form tuberculosis in the study area. Furthermore, the average incidence rate of all form TB in the present study (144/100,000) is also lower than the 164/100,000 recent WHO estimate for Ethiopia [4]. Similarly, the average incidence rate of smear positive pulmonary TB observed in this study (27/100,000) is lower than the 214/100,000 finding from central part of the country [27]. This continuous and significant decline in the incidence and prevalence rate of tuberculosis in the study area might be attributed to the effectiveness of TB/HIV prevention and control programs in the country particularly in the study area.

Furthermore, in the present study treatment success rate (TSR) of all form TB patients was 93%, which is slightly higher than the 90% global target set by WHO [3]. This result is also high when compared to previous TSR reports in the country and neighboring states [15–19, 28]. In line with our finding, another study from northwest Ethiopia reported a TSR of 94.8% which is comparable with this study result [29]. The possible explanation for the high treatment success rate in the present study might be attributed to the expansion of tuberculosis diagnostic and treatment services to the lowest health facility levels where the community lives. Moreover, the role of health posts in active community TB surveillance, tuberculosis treatment defaulter tracing and TB awareness creation in the community is also another possible reason for the high TSR in the area. However, despite the high TSR in the present study, the overall cure rate was 79.2%, which is relatively lower than the 85.5% report from northern Ethiopia [30] and the 84.7% report from northeast Ethiopia [17]. Furthermore, despite the national tuberculosis guideline recommendation to have a sputum smear examination at 2, 5 and 7 months of anti-TB initiation, this study found that nearly 20% of the cases as they have completed their anti TB courses without any confirmed bacteriological result. This is largely due to the presence of none diagnostic health facilities in the study area.

The average death rate in this study was nearly 3%. This is low when compared to previous study reports of 10.5%, 6.3% and 7.4% in different parts of the country [7, 14, 27]. The reason might be attributed to the consorted efforts in the implementation of TB/HIV collaborative activities in the study area [6].

### Conclusion

In the study area, the magnitude of tuberculosis had decreased over the periods reviewed. The proportion of extra pulmonary tuberculosis is high. Therefore, we recommend expansion of culture and chest X-ray diagnostic services to capture EPTB and PTB**−** in the study area. Further research is also warranted to investigate the reasons for the high proportion of EPTB cases in the study area.

## Limitations

Finally, our study is not without limitations. Firstly, we are unable to compare the data obtained from Zonal Health Management Information System (HMIS) database with the reporting health facilities tuberculosis register. However, we have cross-checked the consistency of data between HMIS database and zonal paper based TB reports with zonal HMIS and TB program officers. Secondly, it would have been more representative of the general population if it was a community-based study, instead of a facility-based survey. However, since presumptive tuberculosis case is under active public health surveillance in Ethiopia and partners are also actively working with government in TB program implementation in the zone, all TB cases had been expected to be captured into the surveillance system in the study periods. Thus, our findings are still significant and relevant in drawing attention to the general population. Thus, the findings of the current study should be interpreted in light of the above limitations.

## Abbreviations

AFB: acid fast bacilli
DOTS: directly observed treatment, short course
EPTB: extra-pulmonary tuberculosis
HIV: human immunodeficiency virus
HMIS: health management information system
PTB: pulmonary tuberculosis
PTB+: smear-positive pulmonary tuberculosis
PTB**−**: smear-negative pulmonary tuberculosis
TB: tuberculosis
Trend χ^2^: chi square for trends
TSR: treatment success rate
WHO: World Health Organization
